# Optimization of Data-Independent Acquisition Mass Spectrometry for Deep and Highly Sensitive Proteomic Analysis

**DOI:** 10.3390/ijms20235932

**Published:** 2019-11-26

**Authors:** Yusuke Kawashima, Eiichiro Watanabe, Taichi Umeyama, Daisuke Nakajima, Masahira Hattori, Kenya Honda, Osamu Ohara

**Affiliations:** 1 Department of Applied Genomics, Kazusa DNA Research Institute, Chiba 292-0818, Japan; ykawashi@kazusa.or.jp (Y.K.); nakajima@kazusa.or.jp (D.N.); 2 Laboratory for Gut Homeostasis, RIKEN Center for Integrative Medical Sciences, Kanagawa 230-0045, Japan; eiichiro.watanabe@riken.jp (E.W.); kenya@keio.jp (K.H.); 3 Department of Microbiology and Immunology, Keio University School of Medicine, Tokyo 160-8582, Japan; 4 Department of Pediatric Surgery, Faculty of Medicine, The University of Tokyo, Tokyo 113-8655, Japan; 5 Laboratory for Microbiome Sciences, RIKEN Center for Integrative Medical Sciences, Kanagawa 230-0045, Japan; taichi.umeyama@riken.jp (T.U.); m-hattori@aoni.waseda.jp (M.H.); 6 Graduate School of Advanced Science and Engineering, Waseda University, Tokyo 169-8555, Japan; 7 Laboratory for Integrative Genomics, RIKEN Center for Integrative Medical Sciences, Kanagawa 230-0045, Japan

**Keywords:** DIA, SWATH, deep proteomics, label-free quantification, single shot, overlaping window DIA

## Abstract

Data-independent acquisition (DIA)-mass spectrometry (MS)-based proteomic analysis overtop the existing data-dependent acquisition (DDA)-MS-based proteomic analysis to enable deep proteome coverage and precise relative quantitative analysis in single-shot liquid chromatography (LC)-MS/MS. However, DIA-MS-based proteomic analysis has not yet been optimized in terms of system robustness and throughput, particularly for its practical applications. We established a single-shot LC-MS/MS system with an MS measurement time of 90 min for a highly sensitive and deep proteomic analysis by optimizing the conditions of DIA and nanoLC. We identified 7020 and 4068 proteins from 200 ng and 10 ng, respectively, of tryptic floating human embryonic kidney cells 293 (HEK293F) cell digest by performing the constructed LC-MS method with a protein sequence database search. The numbers of identified proteins from 200 ng and 10 ng of tryptic HEK293F increased to 8509 and 5706, respectively, by searching the chromatogram library created by gas-phase fractionated DIA. Moreover, DIA protein quantification was highly reproducible, with median coefficients of variation of 4.3% in eight replicate analyses. We could demonstrate the power of this system by applying the proteomic analysis to detect subtle changes in protein profiles between cerebrums in germ-free and specific pathogen-free mice, which successfully showed that >40 proteins were differentially produced between the cerebrums in the presence or absence of bacteria.

## 1. Introduction

The dynamic range of protein expression in biological samples is very wide, and the expression levels of kinases and transcription factors of interest in the fields of biology and medicine are low [1]. The sensitivity and depth of proteomic analysis have been improved through development of mass spectrometry (MS), pretreatment, and liquid chromatography (LC) separation technologies, which have made it possible to analyze lowly expressed proteins, including those of interest [2,3,4,5]. For deep proteomic analysis, multi-dimensional protein identification technology (MudPIT), in which digested peptides are fractionated on strong cation exchange, high-pH C18, and other LC columns followed by LC-MS/MS analysis, is the primary approach used [1,6,7,8,9]. Moreover, by combining MudPIT with isobaric tags, such as tandem mass tag and isobaric tags for relative and absolute quantification, in-depth comparative proteomic analysis is possible [10,11]. As analysis technology advances in this way, analyses of multiple samples, such as clinical specimens, are now required. However, since isobaric tags are expensive and the number of samples that can be compared simultaneously is limited, it is not easy to analyze tens or hundreds of samples. Label-free proteomics using MudPIT are difficult to perform because of problems with fractionation reproducibility and throughput. Additionally, single-shot LC-MS/MS is realistic for proteomic analysis of multiple samples, and the technology required for deep proteomic analysis using single-shot LC-MS/MS is needed. Furthermore, a high-sensitivity system that can also handle trace sample analysis would be ideal.

Although data-dependent acquisition (DDA)-MS has been generally used in single-shot proteomic analysis, analysis by data-independent acquisition (DIA)-MS is now often performed because of its excellent depth of analysis, quantitativeness, and reproducibility [12,13,14,15,16]. Typical DIA-MS uses sequential wide isolation windows to acquire comprehensive MS/MS data [17]. However, DIA-MS still has a problem. When a large number of molecules pass through a wide isolation window resulting in a complex MS/MS spectra, accurate quantification and identification are hindered. In recent years, variable-window DIA (vDIA)-MS [18] and overlapping-window DIA (oDIA)-MS [19] have been developed to reduce this problem. In areas where the MS peaks of peptides are richly detected, vDIA-MS reduces the isolation window width and widens the isolation window width in areas where MS peaks are poor. The advantage of oDIA-MS is that it overlaps the isolation windows and demultiplexes them computationally. This process reduces the complexity of the MS/MS spectra. In addition, software dedicated to DIA analysis, such as DIA-Umpire [20,21], PECAN [22], and EncyclopeDIA [23], have been developed to enable identification of peptides and proteins directly from DIA and perform quantitative analysis. By using these applications, more peptides and proteins can be identified with DIA than with DDA. DIA data analysis can also be performed with commercially available software, such as Scaffold DIA, Spectronaut, and PEAKS Studio, and the data analysis platform can be easily prepared.

The sensitivity of single-shot proteomic analysis can be dramatically improved by running LC-MS/MS at a flow rate of ≤50 nL/min on a miniaturized 25 to 30 μm diameter analytical column [24,25,26,27]. However, a column with a small inner diameter is difficult to handle because it takes a long time to load a sample and is easily clogged, so it is not suitable for measurements on a daily basis. If the 75 μm inner diameter column commonly used in proteomic analysis is affected by low-flow rates, it would be possible to construct a highly sensitive system that can be used on a daily basis.

In the advanced single-shot proteomic analysis using the latest equipment, >6000 and >2500 proteins have been identified from 200 ng and 10 ng samples of mammalian cells, respectively, by using online parallel accumulation-serial fragmentation on a timsTOF pro mass spectrometer [28]. In addition, high-field asymmetric waveform ion mobility spectrometry on a Orbitrap Fusion Lumos Tribrid mass spectrometer identified >8000 proteins by DDA-MS during a 6 h long gradient [29]. In DIA-MS, Muntel et al. detected >10,000 proteins during a 6 h long gradient [30]. Although it is commendable that >10,000 proteins were detected, the 6 h long gradient has low throughput and is difficult to use in multi-sample analyses.

Because there still remain many issues with regards to the use of DIA-MS as a sensitive and conventional proteomic tool, as described above, we aimed to establish a robust DIA-MS system for practical proteomic applications in this study. By optimizing various conditions of nanoLC and DIA-MS analysis, we established a robust system suitable for practical applications without scarifying the sensitivity and the throughput. As a demonstration of the power of this system, we used the developed system to compare the protein expression levels of germ-free (GF) and specific pathogen-free (SPF) mouse cerebellums. In recent years, intestinal bacteria have been reported to be associated with brain diseases, such as neurodegenerative disease [31,32,33,34]. However, most of the researches have been limited to analyzing the correlation between intestinal microbiota and disease, and the complicated molecular mechanisms remain unclear. Here, as a first step towards a comprehensive understanding of the mechanisms underlying these phenomena, we successfully demonstrated that brain protein profiles were actually affected depending on the presence or absence of bacteria by our proteome system.

## 2. Results and Discussion

### 2.1. Evaluation of the Flow Rate of nanoLC-MS/MS

**Figure 1:**
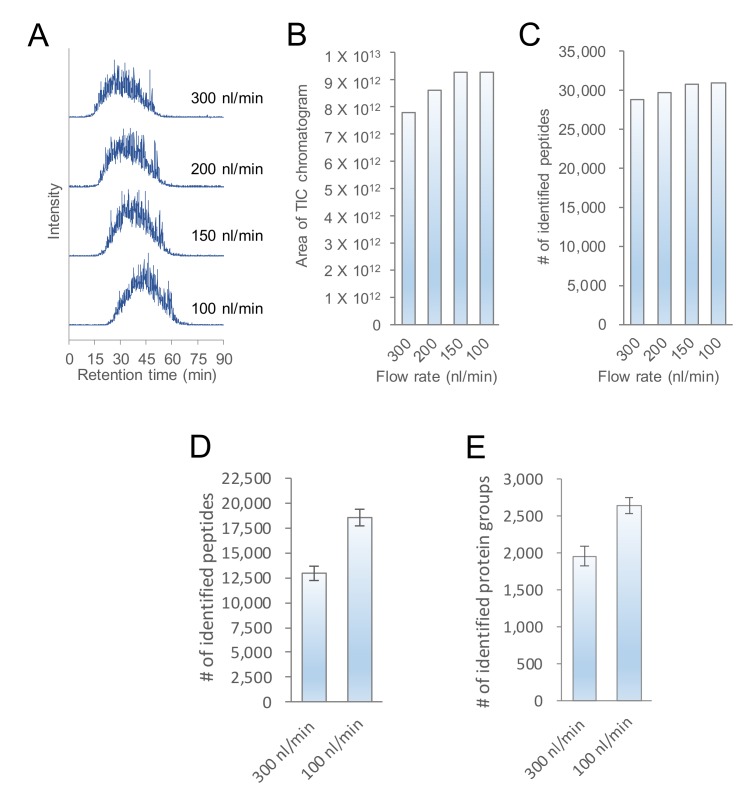
Increased number of proteins and peptides separated and identified by reducing the flow rate of the nanoLC-MS/MS using a 250 mm × 75 μm ID C18 column. (**A**) Total ion current (TIC) chromatograms analyzed by LC-MS/MS at flow rates of 300, 200, 150, and 100 nL/min for 200 ng of HEK293F cell tryptic digest. (**B**) The areas of the TIC chromatograms and (**C**) the number of identified peptides analyzed by LC-MS/MS at the four flow rates in 200 ng of the HEK293F cell tryptic digest. (**D**) The number of peptides and (**E**) protein groups identified by LC-MS/MS at the flow rates of 300 and 100 nL/min in 10 ng of HEK293F cell tryptic digest.

**Figure 2:**
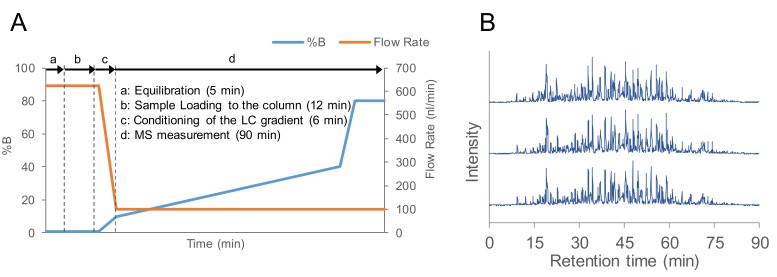
Optimization of the LC program for a gradient flow rate of 100 nL/min. (**A**) LC program optimized for LC-MS/MS over 90 min at a flow rate of 100 nL/min. (**B**) Base peak chromatograms analyzed by LC-MS/MS with the optimized program, measured in triplicate.

In the nanoLC-MS/MS analysis, we used a conventional column with an inner diameter of 75 μm to examine changes in the total ion current (TIC) chromatogram area, and to identify the number of peptides that depended on the flow rate of the nanoLC. In Figure 1A–C, 200 ng of floating human embryonic kidney cells 293 (HEK293F) cell tryptic digest was analyzed at a typical flow rate of 300 nL/min and lower flow rates of 200, 150, and 100 nL/min. The flow rate of 50 nL/min was tried, but the spray was not stable. For this level of flow rate, a 25–30 μm diameter column is typically used, although with the 75 μm diameter column, we considered spray may became unstable due to the decrease in the linear flow rate. As the flow rate decreased, the peak elution times of peptides became slower overall, but the total area value of the TIC chromatogram and number of peptide identifications increased. In the analysis of 10 ng of HEK293F cell tryptic digest, the LC-MS/MS at flow rate of 100 nL/min clearly identified more proteins and peptides than the LC-MS/MS at flow rate of 300 nL/min (Figure 1D,E). Although there had been many reports on higher sensitivity with decreasing flow rate in columns with a narrow inner diameter, we have confirmed that the nanoLC-MS/MS system can be made highly sensitive by lowering the nanoLC flow rate even on columns with an inner diameter of 75 μm. As a result, 100 nL/min, which separated the most peptides, was considered to be the optimum flow rate. However, it took approximately 25 min for the peptide peak to be detected at 100 nL/min. To save time for the gradient, a program was created to start MS measurements after reducing the flow rate to 100 nL/min post-sample loading and increasing mobile phase B to 10% at 6 min (Figure 2A). The total run time, including column equilibration, sample loading, and 90 min of MS measurement time, was about 115 min for one analysis. When the HEK293F cell tryptic digest was analyzed by using these separation conditions, peptide peaks were detected starting from approximately 9 min (Figure 2B). In addition, the chromatograms of the three measurements were similar, which confirmed that the analysis using the improved LC program was reproducible.

### 2.2. Comparison of MS/MS Acquisition Methods by Single-Shot Proteomics

**Figure 3:**
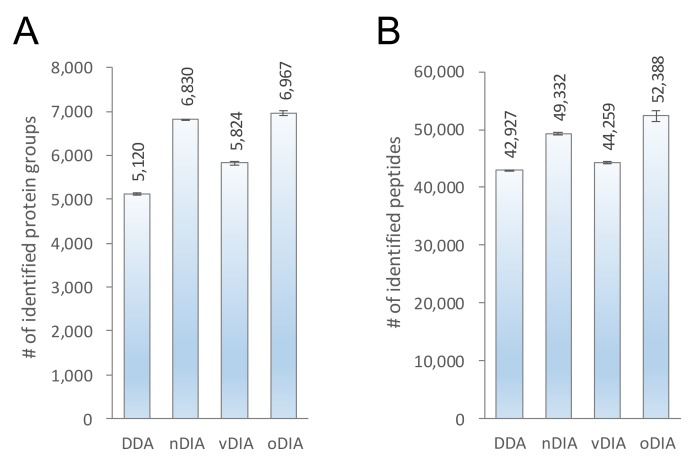
Comparison of data-dependent acquisition (DDA)-MS and three types of data-independent acquisition (DIA)-MS by shotgun proteomics. (**A**) The number of peptides and (**B**) protein groups identified by LC-MS/MS with DDA, normal window DIA-MS (nDIA), variable window DIA-MS (vDIA), and overlapping window DIA-MS (oDIA) in 200 ng of HEK293F cell tryptic digest.

In Figure 3, 200 ng of the HEK293F cell tryptic digest was analyzed by combining the nanoLC program depicted in Figure 2 with each of the four MS/MS acquisition methods. Since DDA-MS and vDIA-MS are suitable for acquiring MS/MS data over a wide *m*/*z* range, the MS/MS measurements were made for *m*/*z* from 350 to 1250. In normal window DIA (nDIA)-MS and oDIA-MS, when the MS/MS acquisition target required a wide *m*/*z* range, the isolation window width was increased. To improve analysis depth and identification accuracy, MS/MS acquisition was performed over the m/z 500 to 860 range where many peptides are detected. In the identification analysis, the obtained MS data were compared against a human protein sequence database (20,431 entries). Three types of DIA-MS were identified to have more proteins and peptides than those identified by general DDA-MS. Among them, oDIA-MS was able to identify the most proteins and peptides, followed by nDIA-MS. Since there were a large number of proteins and peptides identified in oDIA-MS and nDIA-MS, we found that measurement with a narrow isolation window width was important for deep proteomic analysis by in DIA-MS. Furthermore, in oDIA, the effect of reducing the complexity of the MS/MS spectra by computational demultiplexing led to an increase in the number of protein and peptide identifications.

**Figure 4:**
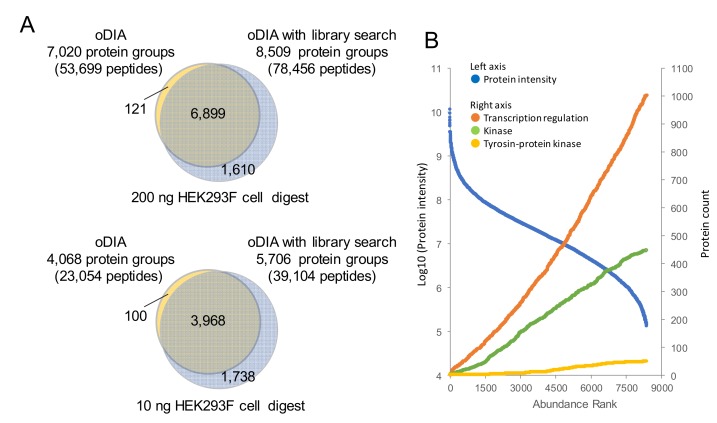
Extending the depth of proteomic analysis by using the chromatogram library created by gas-phase fractionated oDIA-MS. (**A**) Venn diagram showing the overlap of the proteins identified by overlapping window (oDIA-MS) with a library search and without the library search (search against protein sequence database) in 200 and 10 ng samples of HEK293F cell tryptic digest. (**B**) Ranking of HEK293F cell proteins by protein intensity in oDIA-MS with a chromatogram library search (blue dots). Number of transcription factors (orange dots), kinases (green dots), and tyrosine kinases (yellow dots) accumulated in order from the top of the intensity ranking.

Next, chromatogram library searches were performed on the oDIA-MS data that identified the most proteins by protein sequence database searches, and then the number of protein identifications were compared (Figure 4A). The library was created from five gas-phase fractionated oDIA-MS measurements. From 200 ng and 10 ng of HEK293F cell tryptic digest, 7020 and 4068 proteins, respectively, were identified by searching a human protein sequence database. In contrast, 8509 and 5706 proteins from 200 ng and 10 ng of HEK293F cell tryptic digest, respectively, were identified by searching the chromatogram library. In the data from which 8509 proteins were identified, the dynamic range of protein intensity covered 10^5^ (Figure 4B). In addition, the numbers of transcription factors, kinases, and tyrosine kinases, which are considered to be lowly expressed proteins, were 1029, 452, and 50, respectively (Table S1). In a protein sequence database search, the results of our analysis exceeded the results of 6000 and 2500 from 200 ng and 10 ng of Hela digest on a timsTOF pro mass spectrometer that provides the highest analytical performance [28]. Using the library, Muntel et al. previously detected >10,000 proteins from 4 μg of mouse testis tryptic digest by using a combination of DIA with a 6 h gradient [30]. Although our system could not reach 10,000 proteins, we were able to detect 8509 proteins in 90 min from 200 ng of HEK293F cell tryptic digest. Considering the amount and time of analysis, our system gave high performance.

**Figure 5:**
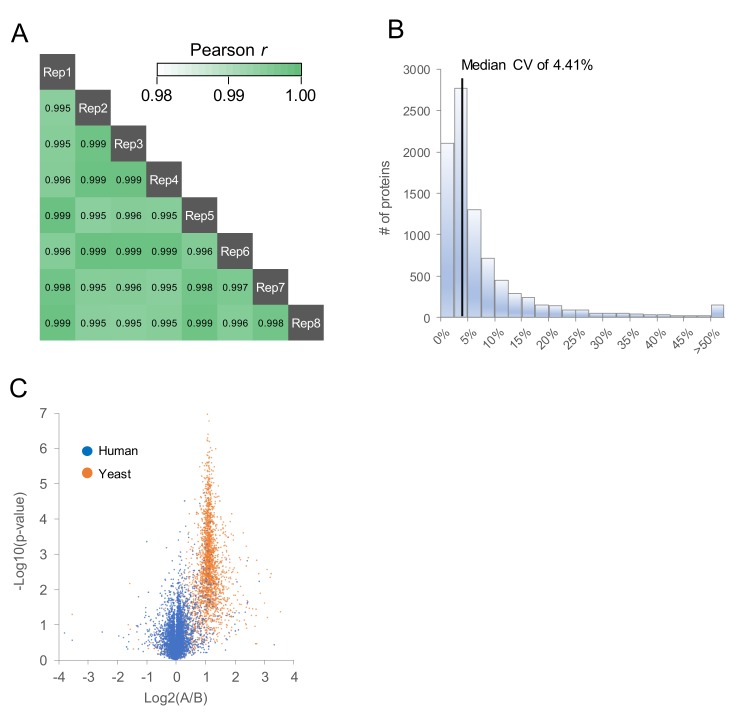
Evaluation of reproducibility and quantitative ability of overlapping window DIA (oDIA-MS) with the chromatogram library search. (**A**) Pearson correlation analysis and (**B**) histogram of CV of protein intensities in eight replicate oDIA-MS runs of 200 ng injections of HEK293F cell tryptic digest. (**C**) Volcano plot against protein intensities obtained by Samples A and B analyzed by oDIA-MS with the library search (each, *n* = 3). Sample A is 200 ng of HEK293F digest and 20 ng of yeast digest. Sample B is 200 ng of HEK293F digest and 10 ng of yeast digest.

To evaluate the reproducibility of oDIA with the library search, the HEK293F cell tryptic digest was measured eight times continuously, and the protein intensities between measurements were compared (Figure 5A,B). Poisson R was ≥0.99 for all comparisons, and the coefficient of variation median was 4.41%, indicating high reproducibility. To investigate the accuracy of comparative quantification, samples of 200 ng of HEK293F cell tryptic digest spiked with 20 ng and 10 ng of yeast digest were analyzed (Figure 5C). On the volcano plot, human protein converged to Log_2_0, and the yeast protein converged to Log_2_1, confirming that the accuracy of comparative quantification was high. We also confirmed that our system performed comparative analysis of protein expression sufficiently. Therefore, by combining nanoLC with the flow rate of 100 nL/min using the 75 μm inner diameter column and narrow oDIA with the library search, we succeeded in constructing a new proteome analysis system that is easy to handle with high sensitivity, and enables in-depth proteome-coverage.

### 2.3. Proteomic Analyses of GF and SPF Mouse Cerebrums

**Figure 6:**
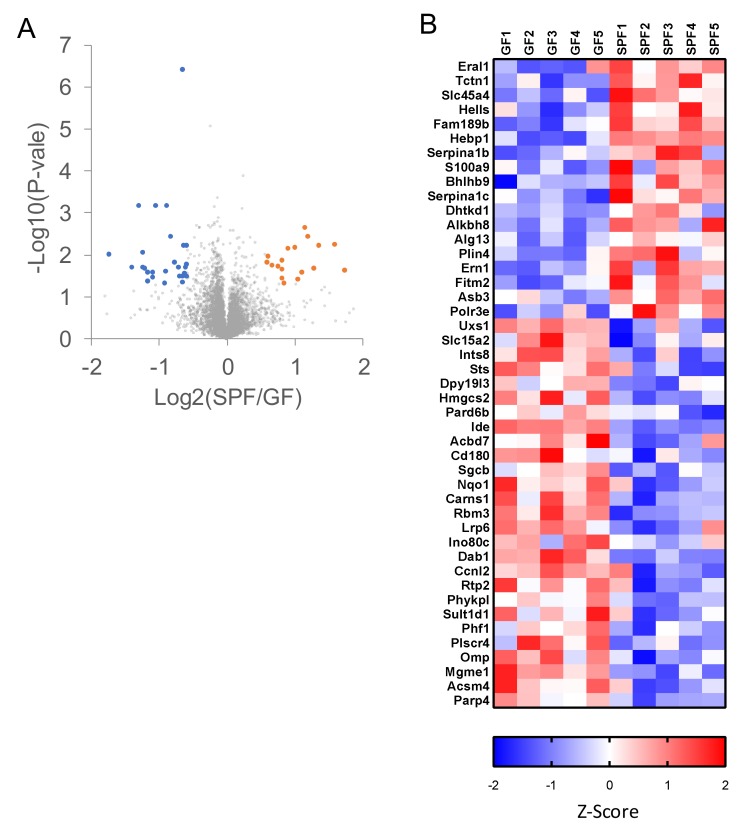
Comparison of cerebrum proteins in germ-free (GF) and specific pathogen-free (SPF) mice by oDIA-MS with the chromatogram library search. (**A**) Volcano plot of protein intensities obtained from GF and SPF mice cerebrums (each, *n* = 5) analyzed by oDIA-MS with the library search. The orange dots (upregulated) and blue dots (downregulated) are proteins (>1.5 fold change and *p* < 0.05) that differed between the two groups. (**B**) Heatmap of proteins (>1.5 fold change and *p* < 0.05) that differed between the two groups.

To demonstrate the power of our system, we performed proteomic analyses of GF and SPF mouse cerebrums by using oDIA with the library search (each, *n* = 5). We expected that a highly sensitive and reproducible protein profiling system would be needed because the presence or absence of bacteria was unlikely to have a significant effect on brain proteins. The library was created by analyzing GF and SPF mouse cerebrum pooled samples by gas-phase fractionated oDIA-MS five times. In Figure 6, the protein expression levels of the GF and SPF mouse cerebrums were compared by making volcano plots and heatmaps. Altogether, 7868 protein groups were identified, with an increase of 18 proteins and a decrease of 27 proteins in the SPF mouse cerebrums. A total of 45 cerebral proteins changed; we observed differences in the expression levels of transcription factors such as *Ino80c*, *Phf1*, *Ccnl2*, *Ern1*, and *Hells* between GF and SPF. Our system was demonstrated to be powerful for comparative analysis of low-expressing proteins. Furthermore, *S100a9* [35,36], *Bhlhb9* [37,38], *Hmgcs2* [39,40], *Ide* [41,42], *Nqo1* [43,44], *Carns1* [45,46], *Rbm3* [47,48], and *Dab1* [49,50] in the altered proteins were reported to be associated with neurodegenerative diseases. *S100a9* related to the promotion of the neurodegenerative diseases decreased, and *Hmgcs2*, *Ide*, *Nqo1*, *Carns1*, *Rbm3*, and *Dab1* related to suppression of the neurodegenerative diseases, other than *Bhlhb9*, increased in GF mouse cerebrums. Considering the alteration of those proteins, GF mice may be less likely to develop neurodegenerative diseases. It has been reported that symptoms of Parkinson’s disease were reduced in GF Parkinson’s disease model mice [51], which is consistent with our findings in this study. Although we did not determine which bacteria in SPF mouse affected specific proteins, it was newly found in this analysis that proteins in the brain alter depending on the presence or absence of bacteria; moreover, the altered proteins included neurodegenerative disease-related proteins. This is an important step in understanding the relationship between bacteria and the brain.

## 3. Materials and Methods

### 3.1. Cell Culture

FreeStyle™ HEK293F cells (Thermo Fisher Scientific, Waltham, MA, USA) were cultured in a shaking incubator at 37 °C with 8% CO_2_ in serum-free FreeStyle™ 293 Expression Medium (Thermo Fisher Scientific, Waltham, MA, USA) to a cell density of 2 × 10^6^ cells/mL, washed with cold phosphate-buffered saline (PBS), and pelleted.

### 3.2. Animal Study

Eight-week-old female GF and SPF mice used in this study were C57BL/6J mice maintained in RIKEN, Yokohama, Japan. GF and SPF mice were anesthetized with isoflurane and transcardially perfused with PBS for 5 min. After the mice were sacrificed by cervical dislocation, their brains were extracted and separated into the cerebrum and cerebellum. These specimens were immediately preserved in liquid nitrogen for proteomic analysis. Animal experiment was approved by the research ethics committee at the RIKEN Yokohama Institute. The approval number was 2018-7(4) and the date of approval was 30 Aug 2019.

### 3.3. Sample Preparation for Proteomic Analysis

The sample was precipitated in acetonitrile (ACN) containing 0.1% trifluoroacetic acid (TFA) by using a water bath-type sonicator (Bioruptor UCD-200; SONIC BIO Co., Kanagawa, Japan) on the high setting for 5 min in 30 s on/30 s off cycles, followed by centrifugation at 15,000× *g* for 15 min at 4 °C to remove the supernatant. The precipitate was extracted in 0.5% sodium dodecanoate and 100 mM Tris-HCl, pH 8.5 by using a water bath-type sonicator (Bioruptor UCD-200) on the high setting for 15 min in 30 s on/30 s off cycles. The extracted proteins were measured by using a BCA protein assay kit (Thermo Fisher Scientific) and adjusted to 1 mg/mL with 0.5% sodium dodecanoate and 100 mM Tris-HCl, pH 8.5. The 20 μg protein extract was treated with 10 mM dithiothreitol at 50 °C for 30 min and then subjected to alkylation with 30 mM iodoacetamide in the dark at room temperature for 30 min. The reaction of iodoacetamide was stopped with 60 mM cysteine for 10 min. The mixture was diluted with 150 μL of 50 mM ammonium bicarbonate and digested by adding 1 μg of Trypsin/Lys-C mix (Promega, Madison, WI, USA) overnight at 37 °C. The digested sample was acidified with 30 μL of 5% TFA, followed by sonication on the high setting for 5 min in 30 s on/30 s off cycles (Bioruptor UCD-200; Cosmobio Co., Tokyo, Japan). The mixture was shaken for 5 min and centrifuged at 15,000× *g* for 5 min. The supernatant was desalted by using C18-StageTips, followed by drying with a centrifugal evaporator. The dried peptides were redissolved in 3% ACN and 0.1% formic acid (FA). The redissolved peptides were measured by using a colorimetric peptide assay kit (Thermo Fisher Scientific) and transferred to a hydrophilic-coated low-adsorption vial (ProteoSave vial; AMR Inc., Tokyo, Japan).

### 3.4. LC-MS/MS

Peptides were directly injected onto a 75 μm × 15 cm PicoFrit emitter (New Objective, Woburn, MA, USA) packed in house with C18 core-shell particles (CAPCELL CORE MP 2.7 μm, 160 Å material; Osaka Soda Co., Ltd., Osaka, Japan) and then separated by using an UltiMate 3000 RSLC nanoLC system (Thermo Fisher Scientific). In a test of flow rates of 75 to 300 nL/min, peptides were separated by using a 90 min gradient of solvents A (0.1% FA in water) and B (0.1% FA in 80% ACN) comprising 1% B from 0 min and 70% B from 90 min. The program optimized for the flow rate of 100 nL/min is described in Table S2. Peptides eluting from the column were analyzed on a Q Exactive HF-X (Thermo Fisher Scientific) for both DDA-MS and DIA-MS analyses. For DDA-MS, MS1 spectra were collected in the range of 350 to 1250 *m*/*z* at 120,000 resolution to hit an AGC target of 3 × 10^6^. The top 80 precursor ions with charge states of 2^+^ to 5^+^ that exceeded 3.5 × 10^5^ were selected for fragmentation with stepped normalized collision energies of 24, 26, and 28, and MS2 spectra were collected in the range of more than 200 *m*/*z* at 15,000 resolution to set an AGC target of 2 × 10^5^. The dynamic exclusion time was set to 15 s. For variable window DIA-MS (vDIA-MS), MS1 spectra were collected in the range of 345 to 1255 *m*/*z* at 120,000 resolution to set an AGC target of 3e6. MS2 spectra were collected in the range of >200 *m*/*z* at 30,000 resolution with stepped normalized collision energies of 24, 26, and 28 to set an AGC target of 3 × 10^6^. Window patterns in the range of 350 to 1250 *m*/*z* were used as window placements optimized by Skyline (Table S3). For normal (nDIA-MS) and overlapping window (oDIA-MS), MS1 spectra were collected in the range of 495 to 865 *m*/*z* at 120,000 resolution to set an AGC target of 3 × 10^6^. MS2 spectra were collected in the range of >200 *m*/*z* at 30,000 resolution to set an AGC target of 3 × 10^6^. The isolation width was set to 6 *m*/*z* with stepped normalized collision energies of 24, 26, and 28. Normal and overlapping window patterns in 500 to 860 m/z were used as window placements optimized by Skyline (Table S3).

MS data for the chromatogram library were created by using the gas-phase fractionation method. We used five MS ranges (498–574, 570–646, 642–718, 714–790, and 786–862 *m*/*z*), and each was measured by oDIA-MS. MS1 spectra were collected at 120,000 resolution to set an AGC target of 3 × 10^6^. MS2 spectra were collected in the range of >200 *m*/*z* at 60,000 resolution to set an AGC target of 3 × 10^6^. The isolation width was set to 2 *m*/*z* with stepped normalized collision energies of 24, 26, and 28. Window patterns in the ranges of 500 to 572, 572 to 644, 644 to 716, 716 to 788, and 788 to 860 *m*/*z* were used as window placements optimized by Skyline (Table S4) [52].

Raw data files of the LC−MS/MS analyses have been deposited in the ProteomeXchange Consortium (http://proteomecentral.proteomexchange.org) via the jPOST partner repository (http://jpostdb.org) with the dataset identifier PXD016032.

### 3.5. Protein Identification by Searching a Protein Sequence Database

MS files were searched against the human UniProt reference proteome (Uniprot id UP000005640, reviewed, canonical; 20,431 entries). For DDA-MS files, the Proteome Discoverer 2.2 (Thermo Fisher Scientific) search engine was used with Sequest HT and Percolator. The setting parameters were as follows: Enzyme, trypsin; maximum missed cleavage sites, 1; precursor mass tolerance, 8 ppm; fragment mass tolerance, 0.02 Da; static modification, cysteine carbamidomethylation. For DIA-MS files, the Scaffold DIA (Proteome Software Inc., Portland, OR, USA) search engine was used. The setting parameters were as follows: Experimental data search enzyme, trypsin; maximum missed cleavage sites, 1; precursor mass tolerance, 8 ppm; fragment mass tolerance, 0.01 Da; static modification, cysteine carbamidomethylation. The protein identification threshold was a peptide or protein false discovery rate (FDR) < 1%. Peptide quantification was calculated by the EncyclopeDIA algorithm [23] in Scaffold DIA. For each peptide, the four highest quality fragment ions were selected for quantitation. Protein quantification was estimated from the summed peptide quantification.

### 3.6. Protein Identification by Searching a Chromatogram Library

A chromatogram library was generated by searching MS data in the library against the human UniProt reference proteome (Uniprot id UP000005640, reviewed, canonical; 20,431 entries) using Scaffold DIA (Proteome Software Inc., Portland OR, USA). The setting parameters were as follows: Experimental data search enzyme, trypsin; maximum missed cleavage sites, 1; precursor mass tolerance, 6 ppm; fragment mass tolerance, 0.006 Da; static modification, cysteine carbamidomethylation. The peptide identification threshold was a peptide FDR < 1%.

For MS data analysis using the chromatogram library, the Scaffold DIA (Proteome Software Inc) search engine was used. The setting parameters were as follows: Experimental data search enzyme, trypsin; maximum missed cleavage sites, 1; precursor mass tolerance, 8 ppm; fragment mass tolerance, 0.01 Da; static modification, cysteine carbamidomethylation. The protein identification threshold was a peptide or protein FDR < 1%. Peptide quantification was calculated by the EncyclopeDIA algorithm in Scaffold DIA. For each peptide, the four highest quality fragment ions were selected for quantitation. Protein quantification was estimated from the summed peptide quantification.

## 4. Conclusions

To achieve high-sensitivity proteomic analysis, we first optimized the nanoLC conditions. We observed that the nanoLC, at a flow rate of 100 nL/min even on a 75 μm column, gave satisfactory results and constructed an LC program for analysis at 100 nL/min. By using the constructed LC program, we tested various MS acquisitions, and oDIA was able to observe the most proteins. We analyzed 200 ng and 10 ng of HEK293F and detected 8509 and 5706 proteins, respectively, by oDIA with the library search. In addition, the reproducibility and accuracy of comparative quantification in oDIA with the library search were confirmed. We succeeded in constructing a high-performance single-shot proteomic analysis system under conditions that can be adapted to routine measurements with 75 μm columns and an analysis time of 90 min. As a demonstration of the power of our system, we performed proteomic analyses of GF and SPF mouse cerebrums and discovered that >40 cerebrum protein levels were changed depending on the presence of bacteria.

Since our system is easy to handle and can observe many kinases and transcription factors related to the cause of the disease, it is expected to provide information useful for elucidating the disease mechanism by applying it to the analysis of clinical specimens such as tissues and cells. In addition, this system is highly sensitive, so we believe it is also suitable for searching biomarkers using exosomes that are collected only in trace amounts from serum and plasma.

## Supplementary Materials

Supplementary materials can be found at https://www.mdpi.com/1422-0067/20/23/5932/s1.

## Abbreviations

MS	Mass spectrometry
DIA	Liquid chromatography
HEK293F	Floating human embryonic kidney cells 293
DIA	Data-independent acquisition
DDA	Data-dependent acquisition
CV	Coefficients of variation
MudPIT	Multi-dimensional protein identification technology
TIC	Total ion current
nDIA	Normal-window data-independent acquisition
vDIA	Variable-window data-independent acquisition
oDIA	Overlapping-window data-independent acquisition
GF	Germ-free
SPF	Specific-pathogen-free
